# Stable Longitudinal Methylation Levels at the CpG Sites Flanking the CTG Repeat of *DMPK* in Patients with Myotonic Dystrophy Type 1

**DOI:** 10.3390/genes11080936

**Published:** 2020-08-13

**Authors:** Mathis Hildonen, Kirsten Lykke Knak, Morten Dunø, John Vissing, Zeynep Tümer

**Affiliations:** 1 Kennedy Center, Department of Clinical Genetics, Copenhagen University Hospital, Rigshospitalet, 2600 Glostrup, Denmark; mathis.hildonen@regionh.dk; 2 Department of Neurology, Copenhagen University Hospital, Rigshospitalet, 2100 Copenhagen, Denmark; kirsten.lykke.knak@regionh.dk (K.L.K.); john.vissing@regionh.dk (J.V.); 3 Department of Clinical Genetics, Copenhagen University Hospital, Rigshospitalet, 2100 Copenhagen, Denmark; morten.dunoe@regionh.dk; 4 Department of Clinical Medicine, Faculty of Health and Medical Sciences, University of Copenhagen, 2200 Copenhagen, Denmark

**Keywords:** epigenetics, genetics, trinucleotide, repeat, inheritance, blood, muscle, biomarker

## Abstract

Myotonic dystrophy type 1 (DM1) is an autosomal dominant multisystem disorder mainly characterized by gradual muscle loss, weakness, and delayed relaxation after muscle contraction. It is caused by an expanded CTG repeat in the 3′ UTR of *DMPK*, which is transcribed into a toxic gain-of-function mRNA that affects the splicing of a range of other genes. The repeat is unstable, with a bias towards expansions both in somatic cells and in the germline, which results in a tendency for earlier onset with each generation, as longer repeat lengths generally correlate with earlier onset. Previous studies have found hypermethylation in the regions flanking the repeat in congenital onset DM1 and in some patients with non-congenital DM1. We used pyrosequencing to investigate blood methylation levels in 68 patients with non-congenital DM1, compare the methylation levels between the blood and muscle, and assess whether methylation levels change over time in the blood. We found higher methylation levels in the blood of DM1 patients than in healthy controls and especially in the patients who had inherited the disease allele maternally. The methylation levels remained relatively stable over time and are a strong biomarker of the disease, as well as of the maternal inheritance of the disease.

## 1. Introduction

Myotonic dystrophy type 1 (DM1) is an autosomal dominant multisystem disorder characterized by a gradual loss of muscle, weakness, and delayed relaxation after muscle contraction (myotonia) [1]. Extramuscular symptoms such as intellectual disability, cataracts, cardiac conduction abnormalities, and endocrine problems may also be present [1]. It is the most common muscular dystrophy with a prevalence between 0.5 to 18.1 per 100,000 [2].

The disease is caused by an expanded CTG repeat in the 3′ UTR (untranslated region) of the *DMPK* gene [3], which results in altered transcription levels of *DMPK* and the nearby genes *SIX5* and *DMWD* [4,5], as well as the transcription of a toxic gain-of-function mRNA. The splice factor MBNL1 binds to the double stranded RNA hairpins formed by the CUG repeats and is sequestered [6], while the splice factor CUGBP1 is upregulated [7]. This leads to the abnormal splicing of a range of other genes [8,9] and contributes to the multisystem phenotype of DM1. The CTG repeat is subject to both intergenerational and somatic expansions, resulting in heterogenous expansion sizes within and among tissues [10], and a tendency towards longer repeats and an earlier age of onset with each successive generation (anticipation) [11].

Although the genetic basis of the disease has been known for more than 20 years, the exact molecular mechanisms that determine the phenotype are poorly understood. The age of onset is very variable, and though there is a correlation between CTG repeat length and disease onset age, the correlation is not absolute [12,13,14]. In addition to this, the rate of disease progression as measured by the muscular impairment rating scale (MIRS) is not correlated to the age of disease onset [15].

Research over the last decade has shown that the variability in clinical phenotype and age of onset can be correlated to sequence interruptions in the CTG repeat [13,16,17,18], as well as DNA methylation of the area surrounding the CTG repeat [19,20]. The hypermethylation of the DM1 locus was, at first, thought to only be present in patients with congenital onset DM1 (CDM1) [21]; however, studies have shown hypermethylation to also be present in patients with non-congenital DM1 [4,19,22] and particularly in patients with repeat interruptions [4,20]. Generally, there are conflicting results regarding the methylation levels of the CpG sites both upstream and downstream of the CTG repeat in patients with non-congenital DM1. Lopez-Castel et al. [23] reported the absence of methylation of the downstream CpG sites and variable levels of methylation of the upstream sites across a range of tissues, while Santoro et al. [4] observed downstream methylation only in patients with repeat interruptions and upstream methylation only in patients with uninterrupted alleles. Barbè et al. [19] reported the hypermethylation of both upstream and downstream CpG sites, mainly in patients with maternal inheritance of the disease allele and only in patients with more than 600 CTG repeats, while Legarè et al. [20] observed variability in the methylation of downstream, but not upstream, CpG sites. While repeat interruptions have been associated with later onset [16,17], lower levels of somatic instability [17,18], and a milder phenotype [18], the association of methylation with clinical features has been more unclear. Hypermethylation has been correlated with earlier disease onset [4,19], muscular and respiratory features [20], and longer CTG repeats [4,19,20] but also with lower levels of somatic instability [20].

The aim of our study was to assess (1) whether elevated methylation in peripheral blood can be detected in a larger number of DM1 patients using a sensitive method, (2) whether blood methylation levels remain stable over time and thus can be used as a predictive biomarker, and (3) whether or not methylation levels in the blood correlate with the methylation levels in the affected tissue (skeletal muscle). To achieve this, we used bisulfite pyrosequencing, a method that can accurately quantify methylation levels with high sensitivity, to study the degree of methylation in the area surrounding the CTG repeat in 68 patients affected by non-congenital DM1 and 73 healthy controls.

## 2. Materials and Methods

### 2.1. Patient Material

The patient cohort consisted of 68 patients (49% female) with non-congenital DM1 who were referred to our department for diagnostic purposes. In 45 patients, the expanded CTG repeat was on the paternal allele, and in nine patients, it was on the maternal allele, while it was unknown for 14 patients. The genetic diagnosis was performed using triplet-primed PCR (TP-PCR) or long-range PCR, and expansions longer than 80 CTG repeats were considered pathogenic. The modal repeat length was known for 24 patients and was determined either by diagnostic PCR or by Southern blotting in cases where the repeat was too long to be determined by PCR.

The initial blood samples were taken at the time of the genetic diagnosis (between 2 and 27 years prior to this study). The mean age at first blood sampling was 31.1 ± 10.8 years (range, 5–59 years). Skeletal muscle biopsies were available from 15 of the patients (at age 32.5 ± 5.3 years at the time of biopsy; range, 27–43 years). A new blood sample was collected from 24 patients—on average, aged 11.8 ± 6.5 years, range 2–26 years—after the first blood sample was collected.

The control cohort consisted of blood samples from 73 healthy individuals (mean age, 31.4 ± 11.5; range, 5–59 years; 49% female). From seven of these individuals, a second blood sample was taken at an average of 27.9 ± 12.5 years after the first sample was available (range, 10–44 years). DNA extracted from skeletal muscle biopsies was available from 12 healthy controls (age, 32.2 ± 11.6; range, 16–50). The muscle biopsies and the blood used as control material were not obtained from the same individuals.

The project has been approved by the National Committee on Health Research Ethics (protocol H-17017556).

### 2.2. Assessment of Repeat Interruptions

Bidirectional triplet-primed PCR (TP-PCR) followed by capillary electrophoresis on an ABI 3730 DNA Analyzer (Thermo Fisher Scientific, Waltham, MA, USA) was used to assess patients for the presence of interruptions in the 3′ and 5′ ends of the CTG repeat. The results were visualized using Peak Scanner 2 (Thermo Fisher Scientific). Repeat interruptions are usually stretches of CCG or CGG repeats towards the 3′ end of the CTG repeat [24,25], although other interruptions such as CTC [24,26], CAG [27], and GCC [28] have also been reported, and interruptions have been observed at both the 5′ and 3′ ends of the repeat [27]. Repeat interruptions can be identified as gaps in the continuous and decreasing pattern of peaks seen when the output of TP-PCR is analyzed (Figure S1). The primer sequences, modifications to the manufacturer’s instructions, and PCR conditions can be found in Table S1.

### 2.3. DNA Extraction and Methylation Analysis

**Figure 1:**
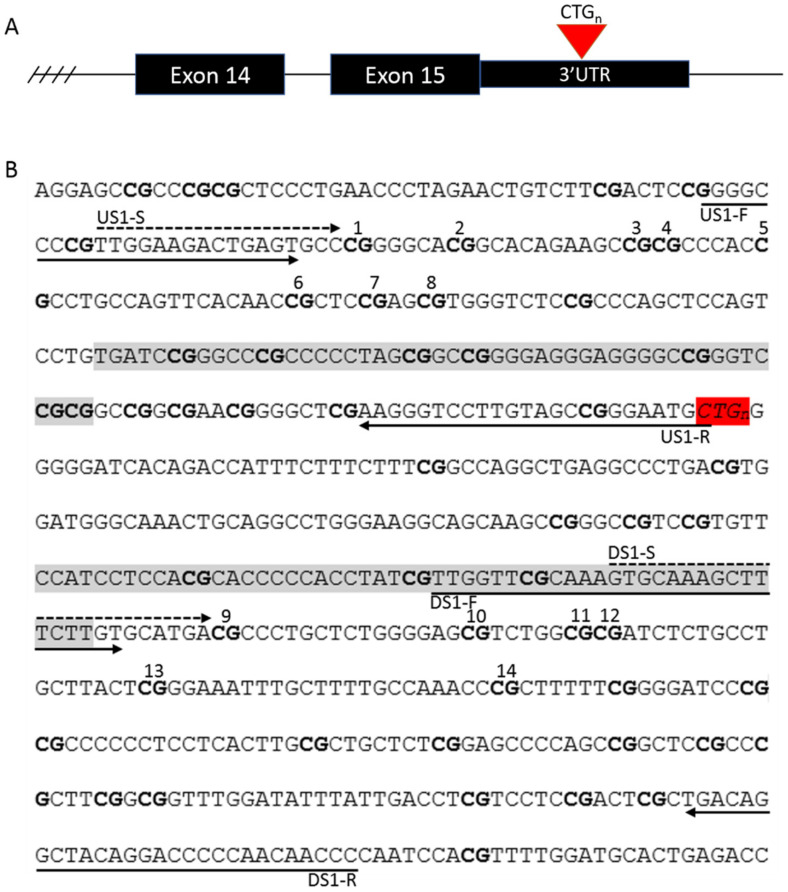
The location of (**A**) the CTG repeat in the *DMPK* gene and (**B**) the primers and the CpG sites investigated in this study. CpG sites 1–8 are located upstream and CpG sites 9–14 are located downstream of the repeat. CpG sites are marked with bold, the CTG repeat is marked with red, and CTCF binding sites flanking the repeat are marked with grey. PCR primers are marked with solid lines, and pyrosequencing primers, with dashed lines.

We used bisulfite pyrosequencing to quantify the degree of DNA methylation at eight CpG sites upstream and six CpG sites downstream of the CTG repeat in the 3′ UTR region of the *DMPK* gene (Figure 1). These regions were chosen as previous studies had associated the methylation of these sites with the age at disease onset [4,19] and the clinical phenotype [20]. Bisulfite conversion was carried out on 200 ng of DNA with an EZ-DNA Methylation-Gold kit (Zymo Research, Irvin, CA, USA), according to the manufacturer’s instructions, with an elution volume of 10 μL. Methylated and non-methylated standards and a no template control (NTC) were included with every conversion. PCR was performed with a Pyromark PCR kit (Qiagen, Hilden, Germany), using 1 μL of bisulfite-converted DNA. The PCR primers were designed with PyroMark Assay Design 2.0 (Qiagen) and were targeted to the regions flanking the CTG repeat (Figure 1). The PCR products were visualized on an agarose gel, and the methylation levels were quantified using a Pyromark Q48 Autoprep (Qiagen) and the Pyromark Q48 software (Qiagen). The primer sequences, modifications to the manufacturer’s instructions, and PCR conditions can be found in Table S1.

### 2.4. Statistical Analysis

Mean methylation levels were calculated as an average of the CpG sites investigated upstream and downstream of the repeat. For statistical analyses the mean upstream and mean downstream values were used, unless specified otherwise.

The Mann–Whitney U test was used to test for methylation differences between patients and controls, and between patients with interrupted and uninterrupted repeats, while the Wilcoxon Signed Rank test was used to test for differences in methylation between blood and muscle samples and between blood samples taken at different times.

Pearson’s correlation was used to test for correlation between methylation and repeat length, correlation between blood and muscle methylation, and correlation between methylation and the patient’s age at the time of sampling. Linear regression was used to assess whether methylation levels were influenced by inheritance (maternal or paternal), repeat interruptions, or sex, as well as to which degree the methylation levels observed in the second blood samples could be predicted by the methylation levels in the first blood sample.

Receiver-operating characteristic (ROC) curves were used to determine whether methylation levels at a specific CpG site could be used as a biomarker for specific characteristics.

All the statistical analyses were conducted using SPSS Statistics (IBM, Armonk, NY, USA) and visualized with R (http://www.r-project.org) and RStudio [29], using the package *ggplot2* [30].

## 3. Results

### 3.1. Repeat Interruptions

The presence of repeat interruptions was detected with bidirectional TP-PCR (3′ TP-PCR and 5′ TP-PCR) followed by capillary electrophoresis to detect gaps in the pattern of amplicon peaks (an example can be found in Figure S1). This revealed repeat interruptions in seven of the 68 patients. All of the interruptions were found toward the 3′ end of the repeat.

### 3.2. Methylation Levels in Initial Blood Samples

**Figure 2:**
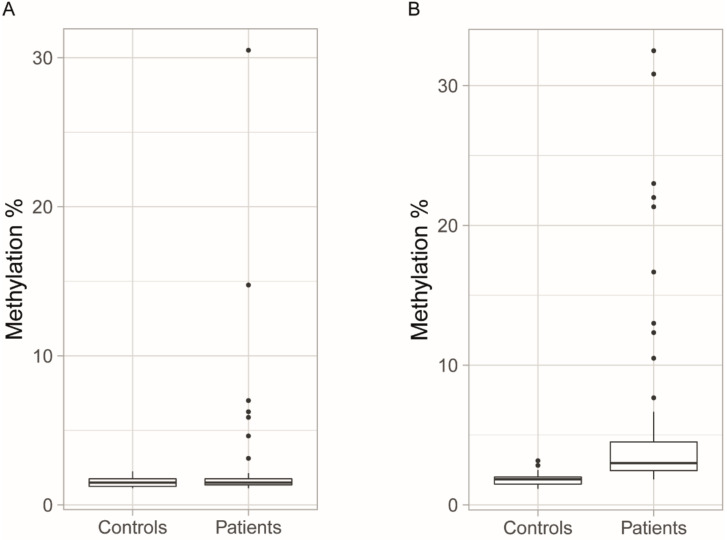
Mean methylation levels of all the CpG sites (**A**) upstream and (**B**) downstream of the CTG repeat. In approximately 10% of the patients, the upstream CpG sites were hypermethylated compared to in the control group, while the methylation levels of downstream CpG sites were significantly higher in patients than in controls. Boxplots indicate medians and quartiles, and the dots indicate the levels of methylation for patients who are outliers.

In this study, we investigated the methylation levels of 14 CpG sites within the DM1 locus. Eight CpG sites were upstream (CpG1–CpG8) of the CTG repeat (upstream CpG sites). Six CpG sites were downstream (CpG9–CpG14) of the CTG repeat (downstream CpG sites). The mean methylation values of the CpG nucleotides were calculated for the eight upstream and six downstream CpG sites and designated as upstream or downstream methylation, respectively (the methylation data for individual patients and controls, and for each CpG site can be found in Table S2). When we investigated the methylation of the upstream and downstream CpG sites of the initial blood samples from 68 patients and 73 control individuals, there was no significant difference between the control group and the non-methylated standard (*p* = 0.958 for upstream CpG sites; *p* = 0.542 for downstream CpG sites). By contrast, both downstream and upstream CpG sites showed variable average methylation in the patients (Figure 2). However, only the downstream sites showed a significant methylation difference in the patients compared to the control group (*p* < 0.0001).

**Figure 3:**
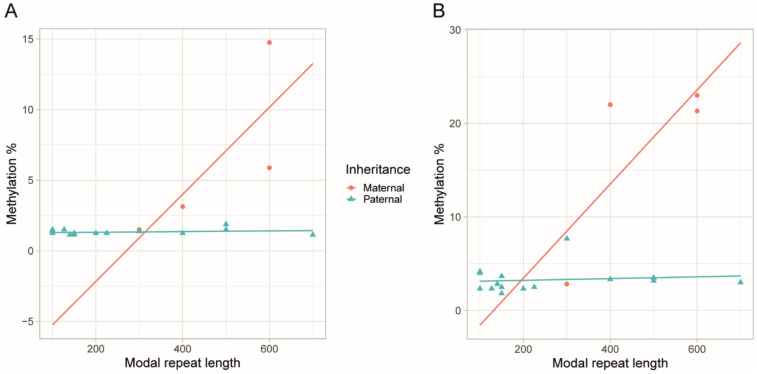
The scatter plots with regression lines show the relationship between the modal repeat size and the methylation levels of the CpG sites of the patients for whom we knew the parental origin of the disease allele. For neither the upstream (**A**) nor downstream (**B**) CpG sites there was a correlation between the modal repeat length and the methylation levels in the patients who had inherited the disease paternally. As the number of the patients who had inherited the maternal disease allele was too low (*n* = 4), it was not possible to make a confident statistical analysis.

There was a positive correlation between modal allele length and methylation levels at both upstream (*r* = 0.476, *p* = 0.019) and downstream (*r* = 0.548, *p* = 0.006) CpG sites (*n* = 24). However, no correlation between the modal allele length and methylation could be observed in the patients (*n* = 15) who had inherited the paternal disease allele (upstream *p* = 0.438; downstream *p* = 0.660; Figure 3). We did not perform this correlation analysis on the patients who had inherited the maternal disease allele, as there were only four patients with known allele lengths among this group. Downstream methylation was significantly higher in the patient group with repeat interruptions (avg. 8.3 ± 6.9% vs. 5.1 ± 6.4%, *p* = 0.027), yet linear regression did not show any significant relationship between repeat interruptions and downstream methylation levels (*p* = 0.223). Upstream methylation was not different in the patients with repeat interruptions compared to in those with uninterrupted repeats (*p* = 0.148). There was a negative correlation between the age at the time of the sampling and the downstream methylation levels (*r* = −0.295, *p* = 0.015), whereas no correlation between the sex of the patients and the methylation levels of either the upstream (*p* = 0.261) or downstream (*p* = 0.718) CpG sites were found.

**Figure 4:**
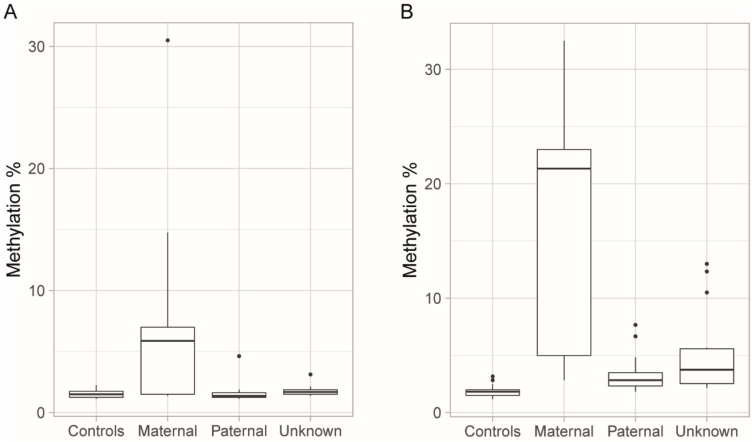
Methylation levels grouped by maternal, paternal, or unknown inheritance. (**A**) Increased methylation of the upstream CpG sites was mainly observed in patients who had inherited the disease allele maternally, while the methylation levels of the patients who had inherited the allele paternally were comparable to those of the controls. (**B**) Increased methylation of the downstream CpG sites was observed in the majority of patients compared to controls, with higher degrees of methylation seen in the patients who had inherited the disease allele maternally. Boxplots indicate medians and quartiles, and the dots indicate the levels of methylation for patients who are outliers.

We used linear regression to determine the relationship between parental transmission of the disease allele and methylation levels in the 54 patients for whom parental inheritance of the disease allele was known. Maternal transmission of the disease was significantly associated with higher methylation at both upstream (*R*^2^ = 0.304, *p* < 0.001) and downstream (*R*^2^ = 0.580, *p <* 0.0001) CpG sites, and increased methylation of upstream CpG sites was present mainly in the patients for whom the disease allele was on the maternal chromosome (Figure 4). At the upstream CpG sites, we only observed increased methylation compared to controls in a single patient with paternal transmission.

### 3.3. Blood Methylation as a Biomarker for DM1 and Disease Inheritance

We used ROC curves to assess whether DNA methylation at specific CpG sites could be used as a biomarker for DM1 and DM1 inheritance. For the assessment of methylation as a biomarker for DM1, the blood samples from all the patients (*n* = 68) and controls (*n* = 73) were included, while for the assessment of methylation as a biomarker of maternal inheritance, the patients with known parental transmission of the allele were included (*n* = 54).

The upstream CpG sites were not useful as biomarkers of DM1, with only CpG1 reaching statistical significance (*p* = 0.05) and an area under the curve (AUC) of 0.60. The downstream sites were better biomarkers for the disease, with the highest AUC found for CpG14 (0.97, *p* < 0.0001), while CpG9–13 ranged from 0.73 to 0.84 (*p* < 0.0001). For predicting whether the disease allele was inherited maternally or paternally, all the CpG sites both upstream and downstream of the repeat were usable (AUC range = 0.79–0.92, *p* < 0.01). The best biomarker of maternal inheritance was CpG11, with an AUC of 0.92 (*p* < 0.0001).

**Figure 5:**
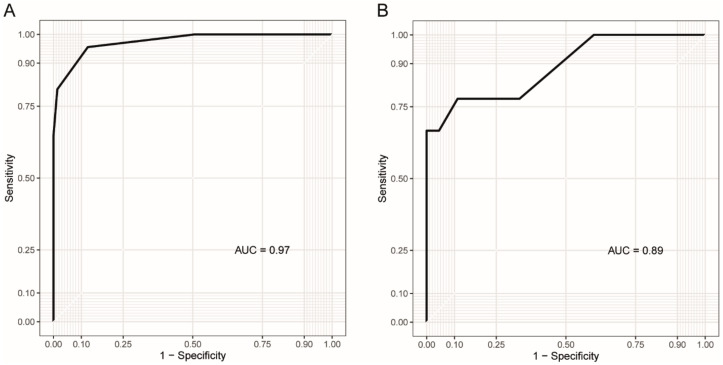
Receiver-operating characteristic (ROC) curves illustrating how methylation at downstream CpG14 can be used as (**A**) a biomarker for DM1 and (**B**) to identify patients with maternal transmission of the disease allele. The *y*-axis shows the rate of true positives, while the *x*-axis shows the rate of false positives. AUC = Area under the curve.

CpG14 was the best biomarker for the combined prediction of the disease and disease inheritance, with an AUC of 0.97 for distinguishing patients from controls and 0.89 for distinguishing maternal from paternal transmission of the allele (Figure 5).

### 3.4. Methylation Levels in Muscle

**Figure 6:**
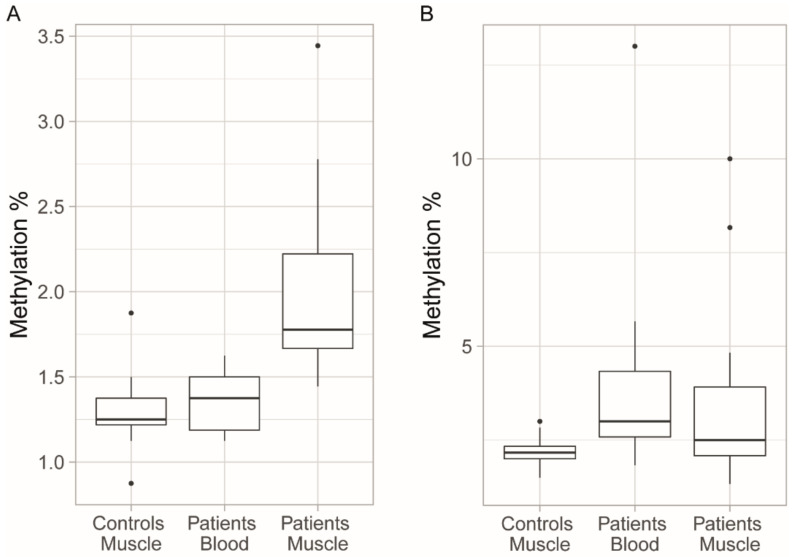
Methylation levels of the CpG sites in the blood and skeletal muscle of DM1 patients compared to controls. (**A**) Increased methylation of upstream CpG sites can be observed in the muscle tissue of patients compared to in the blood of patients and the muscle tissue from controls. (**B**) There was no significant difference in the methylation levels of the downstream CpG sites between the blood and muscle tissue of the patients, and muscle tissue of the controls. There was, however, larger variance in the methylation levels of the downstream CpG sites, and the outliers were values for patients with repeat interruptions. Boxplots indicate medians and quartiles, and the dots indicate the levels of methylation for patients who are outliers.

We compared the methylation levels in the peripheral blood and skeletal muscle in 15 patients. The methylation levels of the upstream CpG sites were slightly higher in the patients’ skeletal muscle than in the patients’ blood (*p* = 0.012) and the muscle controls (*p <* 0.0001), while the methylation levels of the downstream CpG sites were similar in both tissues (*p* = 0.272) and between the patients and muscle controls (*p* = 0.236) (Figure 6). There was no significant correlation between the methylation levels in the muscle and blood, for either upstream (*p* = 0.081) or downstream (*p* = 0.486) CpG sites. Patients with repeat interruptions had higher downstream methylation levels in muscle than patients with uninterrupted CTG repeats (avg. 6.5 ± 3.1% vs. 2.3 ± 0.9%, *p* = 0.006), and repeat interruptions were a strong predictor of downstream methylation levels (*R*^2^ = 0.574, *p* < 0.001). A negative correlation of sample age with methylation was only significant when repeat interruptions were included as a factor in the linear model (*p* = 0.012). When assessing only the 15 patients for whom we had muscle biopsies, we observed a significant relationship between repeat interruptions and downstream methylation levels also in the blood (*R*^2^ = 0.454, *p* = 0.006). As this group was too small, it was not possible to assess the relationship between parental transmission and methylation levels in muscle tissue.

### 3.5. Longitudinal Changes in Methylation Levels in Blood

Longitudinal methylation changes in the blood were assessed in 24 patients. The second blood sample was taken at an average of 11.6 ± 6.3 years after the first blood sample was collected (range, 2–26 years). There was no significant difference in the mean methylation levels of upstream (avg. 1.54% ± 0.70% vs. 1.54 ± 0.63%, *p* = 0.678) or downstream CpG sites (avg. 3.49 ± 2.23% vs. 3.48 ± 1.83%, *p* = 0.757) between the two samples taken at different times. Linear regression analysis showed that the methylation levels in the first blood sample, to a large degree, can be used to predict the methylation levels of the second blood sample at both upstream (*R*^2^ = 0.755, *p* < 0.0001) and downstream (*R*^2^ = 0.755, *p* < 0.0001) CpG sites. There was, however, a positive correlation between the downstream methylation levels and the patients’ ages at the time of sampling (*r* = 0.503, *p* = 0.012). There was no difference or change in the upstream (*p* = 0.581) or downstream (*p* = 0.343) methylation levels between the two blood samples in the longitudinal controls.

## 4. Discussion

Bisulfite pyrosequencing was used to quantify the degree of methylation in the peripheral blood and the skeletal muscle of the patients with non-congenital DM1, as well as to study whether methylation levels in blood were stable or changed over time. Our results show significantly higher methylation levels at the downstream CpG sites in patients compared to controls, despite a few patients exhibiting methylation levels comparable to the mean value of the methylation levels of the controls (Figure 2B). In previous studies of non-congenital DM1-patients, both constitutive hypomethylation [21,23,31] and hypermethylation in a proportion of the patients, primarily those with longer (>600) repeats [4,19,32], have been reported. However, elevated methylation levels downstream of the repeat have been reported in patients with shorter (<500) repeats in a recent study [20], and we report in the present study that the methylation levels of downstream CpG sites are elevated in most DM1 patients compared to in controls, regardless of repeat length and parental transmission. A plausible explanation for the varying and sometimes conflicting results seen in methylation studies of DM1 could be the use of different and sometimes less-sensitive methylation detection methods, especially in earlier studies. In several studies, the patient cohorts were not very large and control groups were small or absent, potentially making it difficult to distinguish low-grade hypermethylation from noise. Upstream methylation was, in our study, limited to a smaller number of patients, mainly those with maternally transmitted disease, as reported previously [4,19]. Repeat interruptions were associated with increased downstream methylation but not with upstream methylation, which is also in line with previous studies [4,20].

The methylation levels of both upstream and downstream CpG sites were relatively stable over time in the blood samples of the patients. This differs from a recent study, where a tendency of decreasing methylation levels over time was observed in DM1 patients [32]. However, the sample size of this study was small (*n* = 3) and only included patients with methylation levels over 10% either upstream or downstream. In the present study, we found that most DM1 patients presented with methylation levels that were higher than those observed in healthy controls, and previous studies have shown that hypermethylation at the DM1 locus in the blood can be linked to earlier disease onset [4,19], lower somatic instability [20], and muscular and respiratory features [20]. Therefore, methylation levels may be used as a prognostic biomarker in some patients. Modifiers of methylation levels, such as disease transmission (maternal/paternal) and repeat interruptions, should be included in the model, especially since patients with repeat interruptions have been shown to present with a milder clinical phenotype [18] and delayed disease onset [13,17], as well as increased levels of methylation. We found in our study that the methylation level of both upstream and downstream CpG sites could be used to indicate which parent the disease allele was inherited from.

We observed a positive correlation between the repeat length and upstream/downstream methylation levels, as reported previously [4,19,20]. When we evaluated the repeat length versus methylation levels among the patients with known parental inheritance of the disease allele, the patients with paternal and maternal inheritance had a tendency to cluster, and the methylation levels in patients with paternal inheritance did not correlate with the repeat length (Figure 3). However, our observations were limited by a number of factors. We had repeat length information for less than half of our patient cohort, and the modal repeat length was measured using different methods. Furthermore, we did not have knowledge of the parental inheritance of the disease allele for every patient. Larger studies may give better insight into this aspect.

We had access to both blood samples and skeletal muscle biopsies from 15 patients, and our study did not reveal any correlation of the methylation levels between the two tissues. We observed elevated methylation levels at the upstream CpG sites in muscle compared to in blood. Longer CTG repeats in muscle compared to in blood have previously been reported [33], and as longer repeats have been associated with elevated methylation levels upstream of the repeat [4,19], it is possible that the higher methylation levels observed in the muscle tissue compared to in the blood could be due to the higher somatic instability in muscle. Our results are consistent with a study by Lopez-Castel et al. [23], which reported the hypermethylation of upstream CpG sites in the muscles of some patients. In this study, the methylation levels of the CpG sites flanking the CTG repeat were investigated in several tissues that included skeletal muscle, but not blood, in five non-congenital DM1 patients [23]. Consistent with our study, there was variation in the methylation profiles of upstream CpG sites between tissues [23]. In our study, we observed the hypermethylation of downstream CpG sites in some patients. In the study cited above, downstream methylation was not observed in the skeletal muscle of the five DM1 patients [23]; however, this could be due to the smaller sample size used.

We observed a correlation between the methylation levels in the initial blood sample and patients’ ages at the time at which the samples were taken for diagnostic purposes. This correlation was also observed in muscle tissue, however, only when repeat interruptions were included as a predictor in the linear model. In many cases, it is likely that the sampling was carried out shortly after the onset of the disease symptoms. However, it was not possible to give a precise age of symptom onset as the patients were referred to our department at different disease stages and, in some cases, as asymptomatic family members of DM1 patients, where the genetic diagnosis was carried out as a predictive test. Most importantly, in several cases, the patients probably did not seek medical assistance with the subtle initial symptoms, and the perception of what constitutes “symptom onset” may vary widely from patient to patient. However, it is still possible that methylation levels can be an indicator of the age of symptom onset, as the diagnostic tests in many cases are likely to be carried out when the disease is suspected. Supporting this, a correlation between methylation levels and age of onset has been reported previously [4,19]. Repeat interruptions have been linked to both higher downstream methylation levels [4,20] and later disease onset [16,17], and it is thus likely that this effect would have to be accounted for if an association between methylation levels and age of onset is to be made. The results of our longitudinal methylation study indicated that the methylation levels do not change with age, which contradicts the idea that age is a modifier of *DMPK* methylation levels.

One of the most important limitations of the present study is the lack of clinical information such as a precise age of onset, the reasons for which are explained above, and a lack of information on the disease severity and rate of progression. Furthermore, for several patients, data on the repeat length were not complete and knowledge about the parental inheritance of the disease allele was lacking. As the repeat length in the blood was determined in a diagnostic setup, we did not have data on the repeat length in the muscle tissue, which could explain the higher upstream methylation in the muscle compared to in the blood in the patients. Other limitations were the lack of information on the type of repeat interruption and investigation of further CpG sites within the DM1 locus. The limitations of this study are mainly due to this study being retrospective, building on an existing diagnostic cohort. Further studies including a broader region and a larger cohort of phenotypically well-described patients and controls are necessary to link the methylation of CpG sites to disease characteristics.

## 5. Conclusions

We have shown in our study that elevated methylation downstream of the *DMPK* CTG repeat is present in a larger proportion of DM1 patients than previously reported and can be used as a biomarker for the disease. The methylation levels both upstream and downstream of the repeat are stable over time in the blood. As methylation levels, previously, have been linked to age of disease onset and clinical phenotype, a blood sample taken early in life could possibly help to predict the age of disease onset, disease progression rate, and severity of the clinical phenotype.

## Supplementary Materials

The following are available online at https://www.mdpi.com/2073-4425/11/8/936/s1. Figure S1: TP-PCR repeat interruptions example; Table S1: Primers and PCR conditions; Table S2: Research data.
