# Supplementary material for: Stable Longitudinal Methylation Levels at the CpG Sites Flanking the CTG Repeat of DMPK in Patients with Myotonic Dystrophy Type 1
Source: Genes (Basel). 2020 Aug 13;11(8):936. doi: 10.3390/genes11080936 (PMC7465187; doi:10.3390/genes11080936)
Supplement: Supplementary file 1 [file genes-11-00936-s001.zip › Figure S1 - TP-PCR repeat interruptions example.docx]

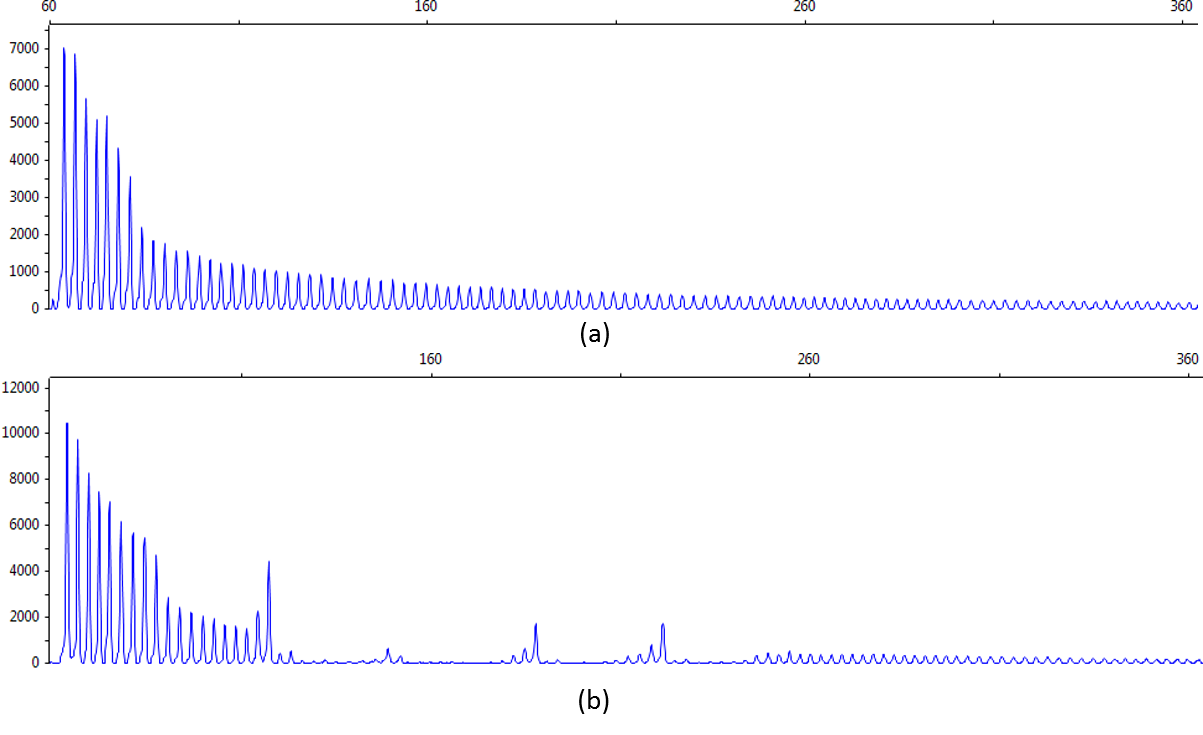


**Figure S1** TP-PCR of the 3’ end of the CTG repeat showing (a) a patient without repeat interruptions (patient 7) and (b) a patient with repeat interruptions (patient 5). Repeat interruptions can be seen as gaps in the otherwise continuous pattern of decreasing peaks. Each peak represents a fragment length, and the peak height represents the number of fragments of a given length. Y-axis=fluorescence intensity, x-axis=fragment length (bp).
